# A comparative study of SIR Model, Linear Regression, Logistic Function and ARIMA Model for forecasting COVID-19 cases

**DOI:** 10.3934/publichealth.2021048

**Published:** 2021-08-26

**Authors:** Saina Abolmaali, Samira Shirzaei

**Affiliations:** 1 Department of Industrial and Systems Engineering, Auburn University, 345 W Magnolia Ave, Auburn, AL 36849, USA; 2 Department of Computer Information System & Analytics , University of Central Arkansas, 201 Donaghey Ave, Conway, AR 72035, USA

**Keywords:** COVID-19, epidemiology, SIR, linear regression, logistic function, ARIMA

## Abstract

Starting February 2020, COVID-19 was confirmed in 11,946 people worldwide, with a mortality rate of almost 2%. A significant number of epidemic diseases consisting of human Coronavirus display patterns. In this study, with the benefit of data analytic, we develop regression models and a Susceptible-Infected-Recovered (SIR) model for the contagion to compare the performance of models to predict the number of cases. First, we implement a good understanding of data and perform Exploratory Data Analysis (EDA). Then, we derive parameters of the model from the available data corresponding to the top 4 regions based on the history of infections and the most infected people as of the end of August 2020. Then models are compared, and we recommend further research.

## 1. Introduction

A pandemic is defined as “an epidemic occurring worldwide, over a very wide area, crossing international boundaries, and usually affecting a large number of people” [1]. Since this is a broad definition that could include seasonal epidemics (which are discarded pandemics), the transmissibility and severity of a disease can be measured to characterize and further describe it. One metric used to measure the transmissibility of a disease is the effective reproduction number (R), which represents the average number of persons infected by one single infectious individual. A measure of severity is the case fatality ratio, which represents the number of deaths caused by the disease. The World Health Organization (WHO) lists nineteen(19) pandemic, epidemic diseases: Chikungunya, Cholera, Crimean-Congo hemorrhagic fever, Ebola virus, Hendra virus infection, Influenza (pandemic, seasonal, zoonotic), Lassa fever, Marburg virus disease, Meningitis, MERS-CoV, Monkeypox, Nipah virus infection, Plague, Rift Valley fever, SARS, Smallpox, Tularaemia, Yellow fever, and Zika virus disease [2]. On March 11, 2020, the WHO declared the novel coronavirus (2019-nCoV) a global pandemic, adding the twentieth disease to this list [3]. On April 25, 2020, the number of confirmed cases reached 2,810,325 and the number of confirmed deaths 193,825, affecting in this way 213 countries, areas, or territories [4].

Some papers discussed the international trade as driver of virus spread [5]–[7]. Some studies discuss SARS-CoV-2 and the corresponding disease [8]–[10] Many researches have discussed the matter as of the effective reproduction numbers [11], [12]. Many researches cover the environmental effect of COVID-19. A study tries to find the connection between weather factors and the spread of virus [13]. Coccia, in his study, discussed the geo-environmental effect on the spread of the COVID-19. Data from North Italy showed a high association between air pollution and the number of infected individuals [14]–[18]. Regarding the spread of the COVID-19 in another work, he discussed geo-ecological determinants of the sped-up dissemination of COVID-19 [19]. Following his work he also developed two indexes which measure the exhibition to confront pandemic dangers by nations, also discussed economic growth of nations [20], [21]. Another study has assessed the connection between ecological contamination determinants and the COVID-19 flare-up in California [22].

Although there are still many questions about this disease, data is being collected and used to learn more about this disease. This study seeks to predict the number of confirmed cases and the number of deaths with the epidemic model and data analytical models. Data analytic have been used in many different areas such as transportation, finance [23] and healthcare. Pandemics have been a topic of interest to several researchers in the data analytic field. Consequently, researchers have been used different models to study the behavior of the data, gain some insight, and draw conclusions. One popular model that is being used is the SIR model. One of the most recent pandemics (before COVID-19) was the H1N1 [24]. According to the Centers for Disease Control and Prevention (CDC), between April 12, 2009, and April 10, 2010, the number of cases reported was 60.8 million and the number of deaths 12,469 in the United States [25]. Ebola (first discovered in 1976) had a recent large outbreak in West Africa (2014–2016). In this significant outbreak, there were 28,652 cases and 11,325 deaths according to the CDC [26].

Chowell et al. [27] discussed the most common modeling approaches used to study and analyze the early spread of an epidemic. These approaches include meta population spatial models, individual-based network models, examining early growth from spatial models (including the SIR model), SIR model with reactive behavior changes, and SIR model with inhomogeneous mixing. The authors identified a gap that requires the incorporation of imperative epidemic features, such as a flexible epidemic growth (from polynomial to exponential dynamics). Mutalik [28] provided a literature review of mathematical models used to predict H1N1 outbreaks. The author included thirty-one (31) articles; nine (9) of them used the SIR model, and the other nine (9) use the SIER model. Other models included: SIS; Compartmental Model; combined model; combined model with SIER – two models only; early exponential growth rate, simple SIER model and complex SIER model, stochastic SIR model; the combination of SIS, SIR, SIER. The author found that the most used mathematical model was the SIER model. The author concluded that a mathematical model along with another secondary model would generate a better prediction.

Zhan et al. [29] used COVID-19 historical data of 367 cities in China and obtained the set of parameters of the augmented Susceptible - Exposed-Infected-Removed (SEIR) model for each city; to create a set of profile codes representing a variety of transmission mechanisms and contact topology. They compared data of the early outbreak of a given population with the complete set of historical profiles. Then, they selected the best-fit profiles and used the corresponding sets of profile codes for predicting the future progression of the epidemic in that population. They applied the method to the data of South Korea, Italy, and Iran. The results showed that peaks of infection cases were expected to happen before the end of March 2020. Moreover, the percentage of the population infected in each city would be less than 0.01%, 0.05% and 0.02%, for South Korea, Italy, and Iran, respectively. In another research Lover and McAndrew [30] used the exponential growth model and epidemiological parameters from the epidemic in Wuhan, China to forecast cumulative infections in the United States. Their forecast results showed that a significant number of infections are undetected, and without considerable non-pharmaceutical interventions, the number of infections are expected to grow exponentially. In another work, Liu et al. [31] used the SEIR model combined with network-driven dynamics to simulate the spread of COVID-19 in the United States accounting for the domestic air traffic occurring amongst the 50 US states, Washington DC, and Puerto Rico. Based on the model predictions for March 14 to March 16, if no containment plans were done, the national epidemic peak could be expected to arrive by early June, corresponding to a daily active count of 7% of the US population. Their results showed that Epidemic peaks were expected to arrive in the Washington and New York states by May 21 and 25, respectively. They also reported that the epidemic progression could be delayed by up to 34 days with a modest 25% reduction in COVID-19 transmissibility via community-level interventions. One work has discussed the prediction of cases in United States using ARIMA and SARIMA models [32]. Another model was implemented by Roosa et al. [33]. They used three phenomenological models to do short-term forecasts in real time. The models had been previously used to perform short-term forecasts for several infectious diseases, including SARS, Ebola, pandemic influenza, and dengue. The generalized logistic growth model (GLM) extended the simple logistic growth model to accommodate sub-exponential growth dynamics with a scaling of growth parameter, p. The Richards model also included a scaling parameter, a, to allow for deviation from the symmetric logistic curve. They also included a sub-epidemic wave model that supports complex epidemic trajectories, including multiple peaks. Based on data up until February 9, 2020, their forecasts agreed across the three models presented to a large extent and predicted an average range of 7409–7496 additional confirmed cases in Hubei and 1128–1929 additional cases in other provinces within the next five days. Models also predicted an average total cumulative case count between 37,415 and 38,028 in Hubei and 11,588–13,499 in other provinces by February 24, 2020. Taking into account the nature of the epidemic disease data is time series, Gupta and Pal [34] applied the ARIMA model to predict the future trends in India. Based on their forecasts generated by the ARIMA model, the number of infected cases in India may go up to 700 thousand in the next 30 days in the worst-case scenario. However, the most optimistic scenario may show the numbers up to 1000–1200. Moreover, the average number of infected cases predicted by the ARIMA model was around 7000 in the next 30 days while the current number was 536. Some studies have discussed the inefficiency of the SIR model and developed a modified SIR model [35]–[37]. A study used the Susceptible-Infectious-Recovered-Dead (SIDR) model and data of the COVID-19 spread in Hubei, China from January 11 to February 10, 2020, to estimate the parameters of basic reproduction number *R*_0_ (2.6 based on confirmed cases and almost 2 considering twenty times the number of confirmed cases and forty times the number of recovered) and per day infection mortality (0.15% considering the second scenario) and recovery rates. The authors also predicted that the epicenter would be on February 29, 2020, with a cumulative number of infected of 45,000–180,000 and a number of deaths of more than 2,700 [38]. Read et al. [39] applied a fitted a deterministic SEIR meta population transmission model with an assumed four (4) days incubation period (based on a SARS approximation) to estimate the *R*_0_, which ranges between 3.6 and 4.0. Moreover, they estimated a transmission rate of 1.07 within Wuhan. The authors estimate that only 5.1% (with a 95% confidence interval) of infections in Wuhan are identified. They also predict more than 190,000 cases by February 4, 2020.

While different models have been proposed [40], [41], it is hard to predict the number of cases because non identifiability in model alignments utilizing the affirmed case information [42]. To compare this study with previous researches, one study has compared the SEIR model with the polynomial model and SEIR showed better results in long term [43]. Another work compares The SIR and the ARIMA model and showed the ARIMA model outperforms the SIR model [44]. This work tries to give a good understanding of the data by providing data visualization in different models. In the next step, we are trying to investigate the modeling and prediction based on each model. We have used logistic function, linear regression, SIR model which is the well-known epidemiologic model, and ARIMA model a time series model to define and predict the number of cases for four countries. These four countries are selected based on the highest number of infected individuals at the period of study. The rest of the study is classified as materials and methods in the second part which describes the data and model used, results and discussion which clarify the outcomes of the models, and conclusion at the end.

## 2. Materials and method

### 2.1. Sample and data

**Figure 1.**
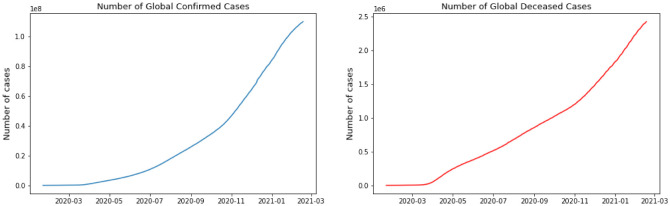
Global number of confirmed cases and deceased cases.

For this research, we have used GitHub data repository managed by Johns Hopkins University which contains daily time series summary tables, including confirmed, deaths and cases infected more than once per day. Daily data of the influenced individuals are very helpful for data scientists. All data are from the daily case report, retrieved from: https://github.com/CSSEGISand Data/COVID-19. The number of global confirmed cases and deaths since January 22 is graphically illustrated in Figure 1. It shows that the expansion begins between March and April 2020. It is important to note that this number includes the reported cases of people who have been tested. No nation knows the actual number of individuals tainted with COVID-19. All we know is the status of the individuals who have been tested. Each of them who has lab-affirmed contamination is considered as a confirmed case. This implies the tallies of affirmed cases rely upon how much a nation really tests and the reliability of results correctness. To decipher any information on the confirmed cases we have to know how much testing for COVID-19 the nation really does. Although these numbers do not exactly reflect the real situation that the world is facing, they still give valuable insight into the behavior of this disease's growth. We broke down our data sets with various EDA techniques and envisioned that information to give an adequate cognizance concerning the flare-up of COVID-19. The top four nations with the most infected patients are the USA, Brazil, India, and Russia.

**Figure 2.**
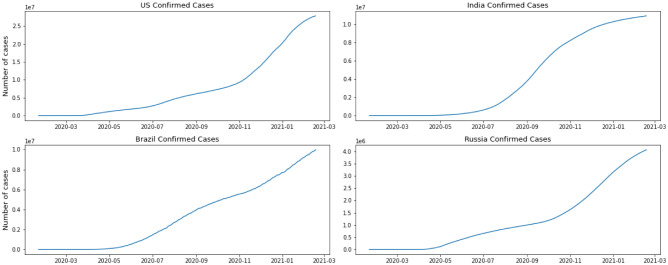
Confirmed cases per country.

**Figure 3.**
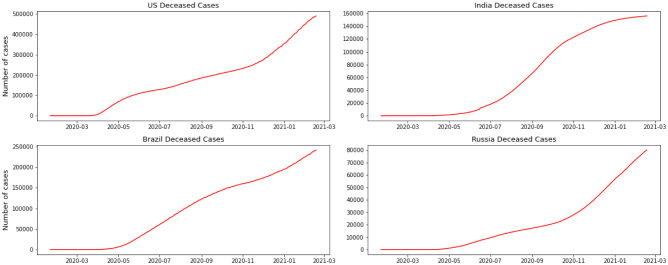
Deceased cases per country.

We have separated the number of confirmed cases and the fatalities to show how Coronavirus is contaminating individuals in each country. This measurement offers two key experiences: initially as a proportion of how sufficient nations are testing; furthermore to assist us with understanding the spread of the infection, related to information on confirmed cases. The positive rate is a decent measurement for how satisfactorily nations are trying because it shows the degree of testing compared to the size of the episode. To have the option to appropriately screen and control the spread of the infection, nations with more boundless flare-ups need to accomplish all the more testing. For classification, regression, or forecast of a specific issue, feature selection techniques can be utilized to discover the highlights that have the most elevated effect on that issue. As indicated by Figures 2 and 3, it doesn't appear that the spread is controlled in any of the referenced nations. As we can see in the depicted charts, the United States has the highest number of infected patients. The figure shows that in almost 4 months of the first case announced in the United State more than 5 million people were infected and after that India has the sharpest rate of infection.

### 2.2. Model and data analysis

**Figure 4.**
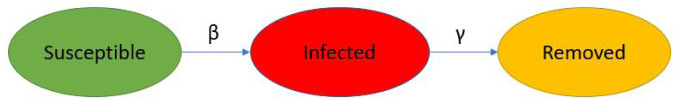
SIR following.

The mathematical modeling of epidemics has been the object of a vast number of studies over the past century [45]. Given the importance of epidemics for life on Earth in general, it is not in the least astonishing that the desire to understand their mechanism has led to the formulation of models which make possible the simulation of events for which laboratory experiments cannot be conducted easily [37]. The reason we have chosen the SIR model is that there is not enough evidence that the patient might not be immune to the disease. Prominent among the mathematical models of epidemics, and great historical importance, is the susceptible–infected- removed (SIR) model initially proposed by Kermack and McKendrick [46]. The model has been defined with three groups of healthy people who are susceptible (S), infected individuals (I), removed individuals either by them being recovered and immunized or by their death (R). Since the number of susceptible, infected, and recovered people may fluctuate over time, the SIR model is dynamic. Flowing from susceptible to infected and then recovered could be showed in the Figure 4.

In this model, the infection rate is *β*, which is the probability of transmitting disease between a susceptible and an infectious individual. *γ* is the recovery rate. *N* is defined as population and is equal to *N* = *S* + *I* + *R*. We can write the SIR model as the following differential equation:

$$\frac{dS}{dt}=-\frac{\beta SI}{N}$$ (1)

$$\frac{dI}{dt}=\frac{\beta SI}{N}-\gamma I$$ (2)

$$\frac{dR}{dt}=\gamma I$$ (3)

To perform the SIR model we have started with 1000 as the number of population. We have used an initial number of infected equal to one and an initial number of removed equal to zero as the data set. Therefore, everyone else is susceptible to infection initially. After taking several tests on the model we have observed that the best combination of the beta and gamma for our data set would be *β* = 3.524 which is a mean number of contacts (sufficient to spread the disease per day that each infected individual has) and also *γ* = 3.45 the infected group that recovers (or dies) during any given day. In this model, we did not consider the influence of immigration because once an epidemic has started, the impact of any additional immigrants is small. The relative impact of an immigrant in the subsequent growth of the epidemic drops geometrically with the number of local infected [47].

Our general surroundings are profoundly muddled. For instance, how an infection spreads, including the novel strand of Coronavirus (SARS-CoV-2) that was distinguished in Wuhan, China, relies on numerous components, among which some of them are considered by the exemplary SIR model, which is somewhat oversimplified and can't contemplate floods in the number of susceptible people. Regression models are utilized to assess or anticipate the target variable based on dependent factors. As we know regression modeling characterizes an influential technique to model and estimate the target variable. For the instance of predicting a continuous amount response variable regression is utilized, while classification is reasonable for foreseeing a discrete class label response. Subsequently, for demonstrating the number of confirmed cases after some time and anticipating future development, regression is thought of. To model the relationship between the response and the explanatory variable we are going to use linear regression. Simple linear regression is a model with a single regressor *x* that corresponds to a response variable *y*. Simple linear regression can be formulated as follow:

$$y=\beta_{0}+\beta_{1}x+\varepsilon$$ (4)

where *β*_0_ is intercept and *β*_1_ is the slope. Both of these parameters are constant. Here *ε* is a random error component.

The logistic equation was initially advanced in 1920 not as an advantageous depiction, yet as a law of development, and was enthusiastically condemned by statisticians and biologists for the resulting decade and a half. However, it endured and rose in an alternate setting as one of the base models of experimental populace biology in the 1930's and 1940's. The move from dismissal to acknowledgment was in no way, easy and was not just because of biologists' progressive acknowledgment of the natural value of the curve. The logistic curve portrays the development of a populace after some time. In its easiest structure it is S-shaped, balanced, and is portrayed with the equation:

$$f(x)=\frac{L}{1+e^{-k(x-x_{0})}}$$ (5)

where

*x*_0_ = sigmoid's midpoint,

*L* = the curve's maximum value,

*k* = the logistic growth rate.

This equation communicates all the more the essential proposal underlying the logistic hypothesis, that the pace of growth diminishes linearly as the population increases. The underlying phase of growth is almost exponential; at that point, as immersion starts, the growth eases back to linear, and at the end, stops the growth. The model can provide a forecast for 3 out of 4 countries closely as the actual data. we have used 200 days to train the model and we have tested the model over the 50 days data after the 200 data.

ARIMA model is a well-known and generally utilized statistical technique for time series forecasting. “Auto-Regressive Integrated Moving Average” is a given time series dependent on its previous values, to forecast future values using the equation. non-seasonal time series with patterns that are not white noises can be modeled by ARIMA. ARIMA model was presented by Box and Jenkins in 1970. ARIMA models have demonstrated proficient ability to create short-term forecasts. This model is based on the idea that variables future value is dependent on the past values of that variable and errors of that variable. This is conveyed as follows:

$$Y_{t}=\varphi_{0}+\varphi_{1}Y_{t-1}+\varphi_{2}Y_{t-2}+...\varphi_{p}Y_{t-p}+\varepsilon_{t}-\theta_{1}\varepsilon_{t-2}-...-\theta_{q}\varepsilon_{t-q}$$ (6)

where,

*Y_t_* is the real value,

*ε_t_* is the random error at time t.

The steps in building ARIMA predictive model consist of model identification, parameter estimation, and diagnostic checking [48]. ARIMA model has been fitted to the data using 180 days as train data and the rest as test data. We can interpret from the charts that the model can be utilized in short-term predictions since the data is changing in long term.

## 3. Results and discussion

Late epidemic behavior identification is important for monitoring and preventing infectious diseases. The effectiveness of predictive models in predicted incidences of infectious disease has proven to be useful. In this stage, we have all the results gathered as the following charts. Four different models were tested for four different countries with the highest number of infected individuals at the time of the study. Different models have depicted different behaviors for each country. The results might change by expanding the time of the study or by changing the time of the study to another section.

**Figure 5.**
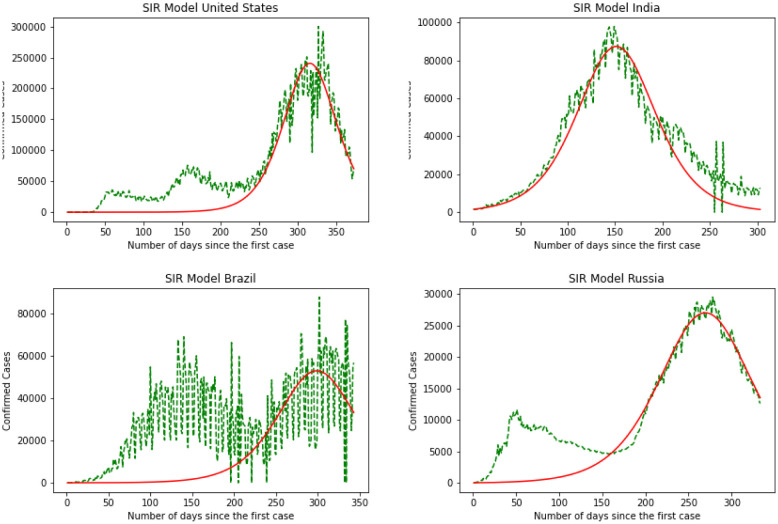
SIR model.

Here we have the results for the SIR model in Figure 5. After calculating the best-fit parameters of the model we plotted the best model for each country. The following figure shows the best possible fit of the data for the United States, Brazil, India, and Russia. The model does not show a good fit for the number of infected individuals. Results indicate that for the United States at early stages the SIR model cannot predict the surge accurately, while it can predict the last surge of the infected individuals. In the case of India, this division is smaller and the graph shows a better fir for India. SIR model is acting the same for Brazil as it did for The United States. Again here we can see the first surge of the infected individuals was not accurately modeled while it was performing better in the second surge. Again the same thing happens for Russia. The results are quantified by using a MSE measure which is mean squared error that is calculating the mean squared difference between the estimated values and the actual value. Here we have MSE(US) = 8127.7, MSE(India) = 3781.9, MSE(Brazil) = 8430.9, MSE(Russia) = 7321.2. At the end of this section, we will compare the MSE results for different methods.

**Figure 6.**
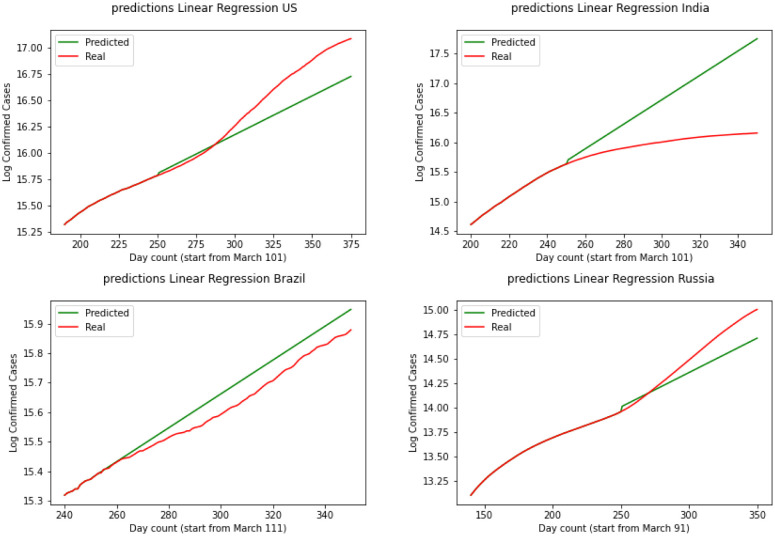
Linear regression.

The graphical and the MSE result shows that the SIR model can not provide a useful early prediction of the epidemic in this case. To improve we have decided to move to regression analysis. Moving forward we have Linear Regression as our second model. Figure 6 demonstrates the results for linear regression of the four countries. Over the test results, we can see that linear regression has performed a better prediction for Brazil over the three other countries. This shows that linear regression can not be used in long term and since this data is nonlinear, a linear model could not explain the data perfectly. By skimming through the charts we can say performs the worst for the United States and India while is performing better for Brazil and Russia. To quantify the error again we have MSE(US) = 3241.2, MSE(India) = 3561.3, MSE(Brazil) = 2658.1, MSE(Russia) = 2601.8. Comparing SIR and linear regression here based on MSE error we can see linear regression is performing a better prediction in the short term.

**Figure 7.**
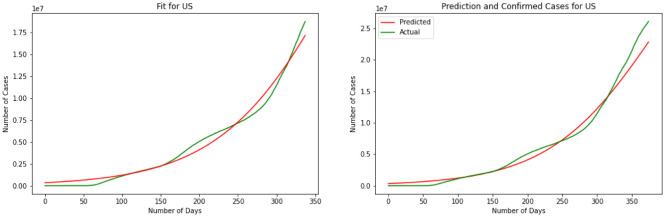
Logistic curve for US.

**Figure 8.**
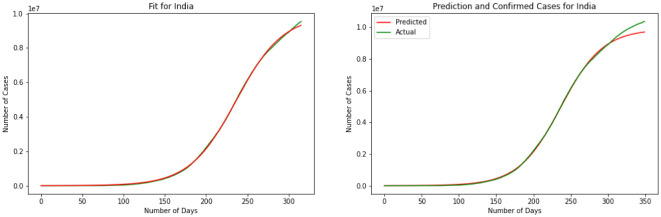
Logistic curve for India.

**Figure 9.**
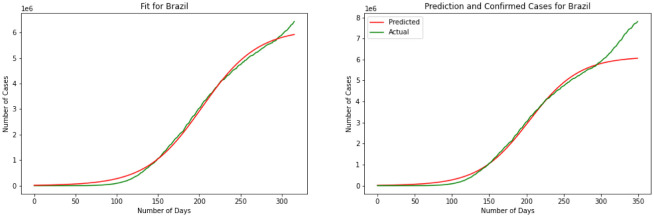
Logistic curve for Brazil.

**Figure 10.**
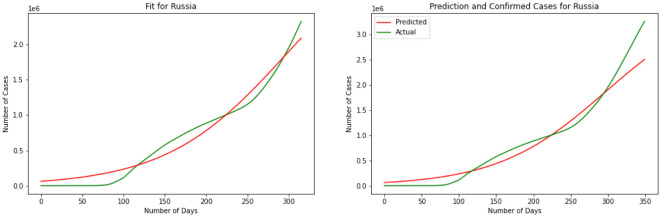
Logistic curve for Russia.

For our third model, we have explained the Logistic Regression in section 2. We have discovered a Logistic Function that is very near the watched COVID-19 information from these four countries. Results for this model are depicted in Figures 7, 8, 9 and 10. The visual examination of the charts says that the model is performing better than the SIR and linear regression. This is to say that the model is visually performing the best for India. To better understand the performance of the model it is better to take a look at the error. Here we have MSE(US) = 678.7, MSE(India) = 631.3, MSE(Brazil) = 731.0, MSE(Russia) = 1501.1. Comparing the MSE we can see model is outperforming in India. Comparing SIR, linear regression, and logistic regression here based on MSE error we can see logistic regression is performing a better prediction in the short term.

**Figure 11.**
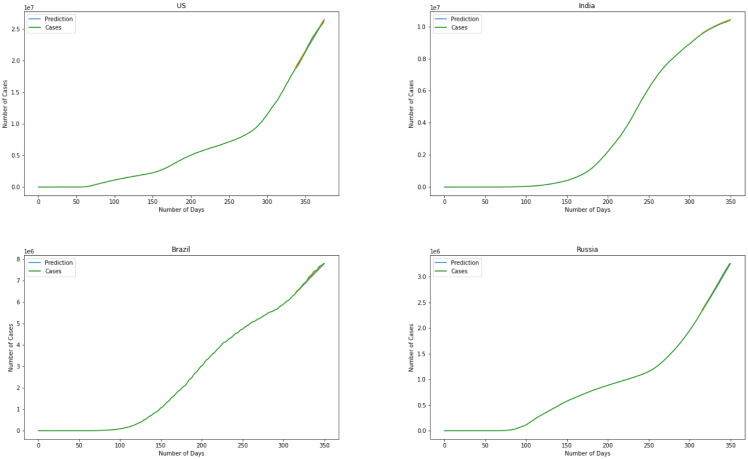
ARIMA model.

Moving on to the last model we have used the ARIMA model to predict the number of infected individuals. Figure 11 is the representation of the model. Albeit further information is required for a more point-by-point forecast, the spread of the infection appears to be modeled precisely. Determining the level of difference, the ARIMA model helps the data remain stationary. This will result in more flexibility for the modeling. Results for the ARIMA model indicate that the model can capture the effect of change in every stage of the data precisely. Errors again is the best representation of the accuracy of the model here. For the ARIMA model we have MSE(US) = 120.2, MSE(India) = 146.8, MSE(Brazil) = 165.4, MSE(Russia) = 102.7. This represents that comparing four models together the ARIMA model managed to present the minimum error for the prediction that means is outperforming the other three models.

## 4. Conclusions

It is necessary to collect and analyze data of a pandemic to assess strategies of intervention, management, and control. This analysis gives a crucial baseline of the characteristics of the transmission and severity of the infectious disease. This study analyzes the behavior of the COVID-19 pandemic in the United States. Moreover, a SIR-based model, Linear Regression, Logistic Regression, and ARIMA model are presented to predict the number of cases and fatalities of this pandemic. Future research could use other models such as variations to the basic SIR model or individual-based network models. Comparisons among these models, in terms of accuracy and magnitude of error, could be made. Results showed that the ARIMA model outperforms all three in the case of prediction. As the case of the limitation of the study was the effect of other parameters like environmental and management effects of the data which cannot be modeled in the series of models presented in this paper. There are some extensions to the sir model that could be considered for further studies. Also, the ARIMA model could be extended to the SARIMA.
